# Prognostic Value of Beclin-1 and HIF-1α in Oral Squamous Cell Carcinoma

**DOI:** 10.1055/s-0045-1806930

**Published:** 2025-07-23

**Authors:** Nadia Attia Radi, Doaa Adel Habba, Seham Ibrahim Hallool, Sulaiman Saeed Alqahtani, Hanaa Mohamed Abd Elsamia

**Affiliations:** 1 Department of Oral and Dental Pathology, Faculty of Dental Medicine for Girls, Al-Azhar University, Cairo, Egypt; 2 Department of Oral Histopathology, Faculty of Dentistry, Sinai University, Arish Branch, ElArich, Egypt; 3 Department of Oral and Maxillofacial Surgery and Diagnostic Sciences, College of Dentistry, Najran University, Najran, Saudi Arabia; 4 Department of Oral and Dental Biology, Faculty of Dental Medicine for Girls, Al-Azhar University, Cairo, Egypt; 5 Department of Oral and Dental Pathology, Faculty of Oral and Maxillofacial Surgery, Zagazig University, Zagazig, Egypt

**Keywords:** autophagy, Beclin-1, HIF1α, OSCC

## Abstract

**Objectives:**

Certain factors that disturb the tumor microenvironment influence the promotion of tumorigenesis. Detecting gene expression at the protein level is highly valuable and complements the histopathological analysis achieved by immunohistochemistry (IHC). Hypoxia-inducible factor (HIF)-1α accomplishes autophagy induction and regulation of autophagy-associated genes. So, this study was carried out to evaluate the tissue protein expression of Beclin-1 and HIF-1α using IHC and correlate their expression with the prognosis of oral squamous cell carcinoma (OSCC).

**Materials and Methods:**

Immunohistochemical evaluation of Beclin-1 and HIF-1α was done in 5 samples of normal oral epithelial tissues and 45 samples of OSCC, which were classified histologically into 15 samples each of well, moderately, and poorly differentiated squamous cell carcinoma.

**Results:**

According to statistics, normal tissue had the highest values for Beclin-1, while poorly differentiated OSCC had the lowest mean area percentage. HIF-1α showed the opposite results. These results indicate that the association of both molecules has a greater role in the transformation from normal to different histopathological grades of OSCC.

**Conclusion:**

The close association between Beclin-1 and HIF-1α identified in the current study confirms hypoxia's critical role in autophagy activation. Moreover, reduced Beclin-1 and elevated HIF-1α expression were significantly associated with the histopathological grading of OSCC, supporting their pivotal role in the development and progression of OSCC.

## Introduction

Oral squamous cell carcinoma (OSCC) is a common head and neck cancer. According to the Global Cancer Observatory, by 2040, the frequency of OSCC will increase by approximately 40%, along with increasing the mortality rate. The multistep growth of diverse genetic changes in squamous cells initiates OSCC. These changes gradually increase the capability of altered cells to proliferate and invade. The heterogeneity of these changes clarifies why tumors at similar sites and clinical stages show major differences in their treatment responses and clinical endings. Owing to its great tendency for local invasion and cervical lymph node metastases, OSCC remains one of the most challenging malignancies to control. 1 2 3 4 5 Histopathological investigation is still the gold standard for most diagnostic and therapeutic decisions despite the recent rise in molecular-based assays. The assessment of gene expression at the protein level has been accomplished by immunohistochemistry (IHC), an internationally available technique that complements histopathological investigation. 6 7

The accurate diagnosis of high-risk OSCC patients can be achieved by applying various prognostic molecular biomarkers, which can provide valuable information. Compared with normal cells, malignant cells may adapt faster to microenvironmental alterations by stimulating multiple stress response pathways and evading antiproliferative and cell death-inducing signals. The process of tumorigenesis, progression, and prognosis in cancer therapy is controlled by tumor hypoxia, which is organized by hypoxia-inducible factors (HIF-1, HIF-2, and HIF-3). The transcription factor known as HIF-1 comprises α and β subunits. It was the first known moderator of human cells' cellular hypoxia response. Under hypoxic conditions, HIF-1α can cause increased anaplastic cell invasion, metastatic spread, and resistance to chemoradiotherapy. The expression of key genes involved in the initiation and progression of autophagosome formation is regulated by HIF-1α. These key genes include *Bcl-2* , *Beclin-1* , *ATG7* , *ATG5* , *ATG9A* , and phosphatidylinositol 3-kinase catalytic subunit type 3. 8 9 10 11

Autophagy is regarded as a double-edged sword in the development of cancer because it can cause cell death when activated for extended periods. Still, it can also cause cell survival at a base level. Beclin-1, which is recorded to a tumor susceptibility locus 150 kb centromeric to *BRCA1* on the human 17q21 chromosome, is a mammalian ortholog of yeast Atg6/vps 30. Beclin-1 indirectly plays a significant role in several biological cellular processes, including endocytosis, development, adaptation to stress, aging, and cell death, and is directly responsible for autophagy initiation. Beclin-1 is considered an essential tumor suppressor gene, so loss or decreased expression resulted in progression, poor prognosis, and chemoradioresistance in several cancers, including hepatocellular cancer. 12 13 14 15 The current study aimed to assess the tissue protein expression of Beclin-1 and HIF-1α through IHC and investigate the correlation between their expression and the prognosis of OSCC.

## Materials and Methods

### Case Selection

The current investigation included four groups of specimens. The first group consisted of normal oral epithelial tissues, including 5 samples from patients undergoing gingivectomy for hyperplastic tissues; 45 cases were from different grades of OSCC, 15 samples each of well, moderately, and poorly differentiated squamous cell carcinoma (GI, GII, GIII, and GIV, respectively). The samples were collected as paraffin blocks from the archives of the Department of Oral Pathology at Al-Azhar University.

### Histopathological Analysis

Histopathologic grading, considering the World Health Organization classification, was performed, and a hematoxylin and eosin stain was applied to re-evaluate all samples included in the study.

### Immunohistochemical Analysis

For the streptavidin-biotin immunohistochemical method, 4-μm-thick sections were mounted on electrically positively charged glass slides. They were deparaffinized by first incubating in xylene for an entire night. They were then rehydrated with ethanol at gradually decreasing concentrations and washed with phosphate-buffered saline (PBS). The application of 3% H _2_ O _2_ for 5 minutes at room temperature was required to block endogenous peroxidase activity. To improve immunoreactivity, tissue sections were placed in a glass jar with 0.01 M sodium citrate buffer (pH 6.0) and then boiled twice in the microwave for 5 minutes each time (antigen retrieval). The slides were rinsed with (pH 7.2) PBS after allowing them to cool. HIF-1α and Beclin-1 antibodies immunohistochemical staining followed the manufacturer's instructions. Both were mouse monoclonal antibodies. Using PBS, the dilution was adjusted at 1:50. HIF-1α (Santa Cruz Biotechnology, United States; Cat. No. SC 53546) and Beclin-1 ([G-11]; Santa Cruz Biotechnology, Cat. No. SC 48381) were utilized to assess the levels of hypoxia and autophagy, respectively, in this research.

The universal kit (DAKO, Denmark) has been used for detection. Wash the slides in PBS for 5 minutes and keep them with secondary antibodies, which are rabbit and mouse sera conjugated with biotinylated goat serum, for 30 minutes. Washing the tissue sections for 5 minutes in PBS followed by developing antigen-antibody visualization by diaminobenzidine in PBS comprising 40% H _2_ O _2_ . For 10 minutes, under tap water, sections were washed, and then Mayer's hematoxylin counterstaining was performed, then the slides were mounted. The current study was approved by the Ethics Committee of the Faculty of Dental Medicine for Girls, Al-Azhar University (Code No. REC-PD-24-07).

### The Analysis of Histomorphometry

Using image analysis of a Leica image analyzer controlled by the Leica Qwin 500 software (Germany), the immunostained positive cells percentage relative to the measured area in every field was assessed to calculate the immunoreactivity for HIF-1α and Beclin-1. The pixels produced by the image analysis program were automatically transformed into actual micrometer unit. The percentage of Beclin-1 and HIF-1α immunostained areas was calculated relative to an ordinary measurement frame of 11,434.9 µm ^2^ using ×200 magnification. For histomorphometric evaluation, 10 fields were gradually collected from each slide section for each patient. The mean values were then determined for each sample.

### Statistical Analysis

The data were displayed with their mean and standard deviation values. Specifically, the analysis of variance (ANOVA) test was used to compare the means of multiple groups. The Tukey's test is used if various comparisons between numerous variables and the ANOVA test are significant. The *p* -value is significant if it is less than or equal to 0.05 ( *p* ≤ 0.05). Pearson's correlation coefficient ( *r* ) test evaluated the correlation between two variables. Version 23.0 of the SPSS program (SPSS Inc., Chicago, Illinois, United States) was used to assess the collected information.

## Results

### Histopathological Findings

**Fig. 1:**
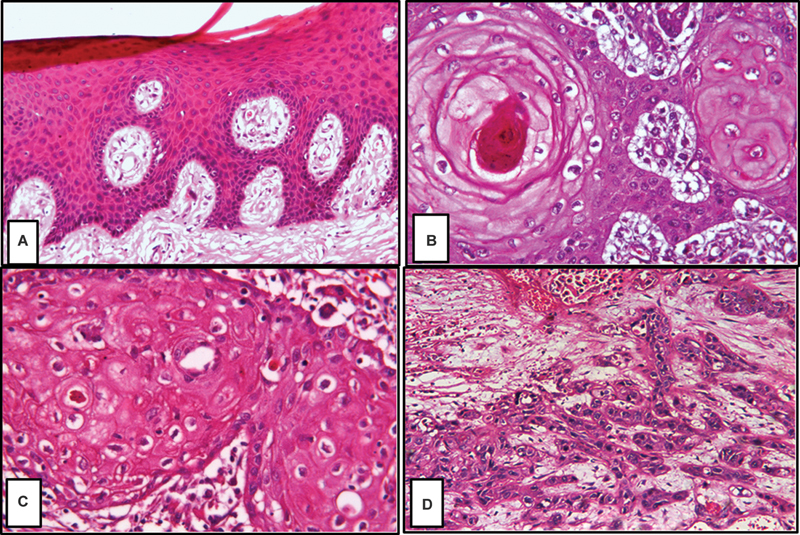
GI, normal gingival tissue showing basal, spinous, granular, and parakeratin layers ( **A** ); GII, WDOSCC showing cell nests and keratin pearls ( **B** ); GIII, MDOSCC showing nests of tumor cells and individual cell keratinization ( **C** ); and GIV, PDOSCC, showing highly anaplastic cells ( **D** ) (hematoxylin and eosin, ×200). MDOSCC, moderately differentiated oral squamous cell carcinoma; PDOSCC, poorly differentiated oral squamous cell carcinoma; WDOSCC, well-differentiated OSCC.

A normal gingival tissue sample exhibited keratinized stratified squamous epithelium of regular thickness. Dense collagen fibers were found in the underlying connective tissue scattered in some areas with fibroblasts and some inflammatory cells. The underlying connective tissue contains invasive nests and islands characterized by large neoplastic cells with distinctive cell membranes. In addition, multiple keratin pearls of different sizes and individual cell keratinization were observed in well-differentiated OSCC (WDOSCC). While in moderately differentiated OSCC, the similarity of the neoplastic cells to their tissue of origin was less pronounced. It was present with minimal keratin and enhanced cellular pleomorphism. There was no similarity between the malignant and mother cells in poorly differentiated grades. Connective tissue was deeply invaded by highly anaplastic cells without keratin formation. Atypical blood vessels and chronic inflammatory cells were also detected ( Fig. 1A–D ).

### Results of Immunohistochemistry

**Fig. 2:**
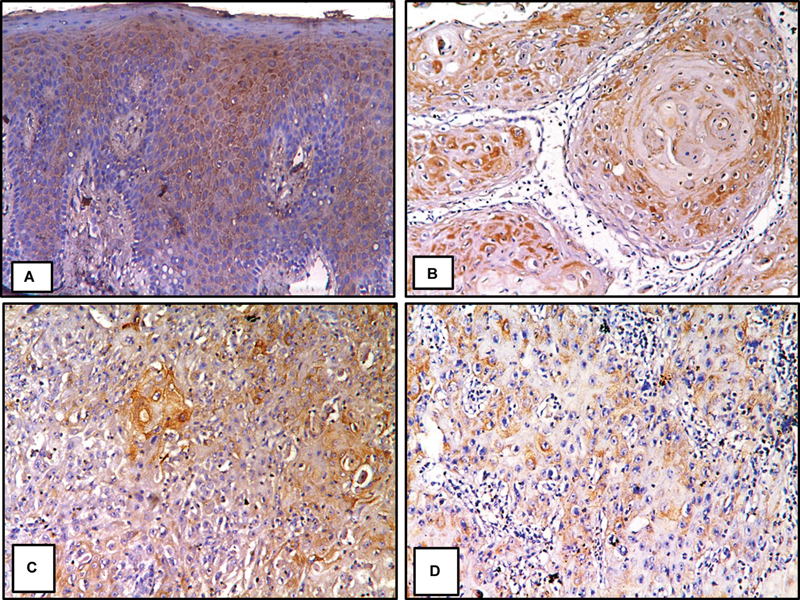
GI, normal oral epithelial tissue showing Beclin-1 immunostaining was detected mainly in the cytoplasm and nucleus of all layers of the epithelium ( **A** ); GII, WDOSCC, showing Beclin-1 immunostaining was seen in the cytoplasm and nucleus of the cancer cells as well as in the keratin pearls ( **B** ); GIII, MDOSCC, showing Beclin-1 immunopositivity was seen in the cytoplasm and nucleus of the malignant cells ( **C** ); GIV, PDOSCC, showing Beclin-1 immunopositivity was seen mainly in the cytoplasm of highly anaplastic cells ( **D** ) (Beclin-1, ×200). MDOSCC, moderately differentiated oral squamous cell carcinoma; PDOSCC, poorly differentiated oral squamous cell carcinoma; WDOSCC, well-differentiated oral squamous cell carcinoma.

**Fig. 3:**
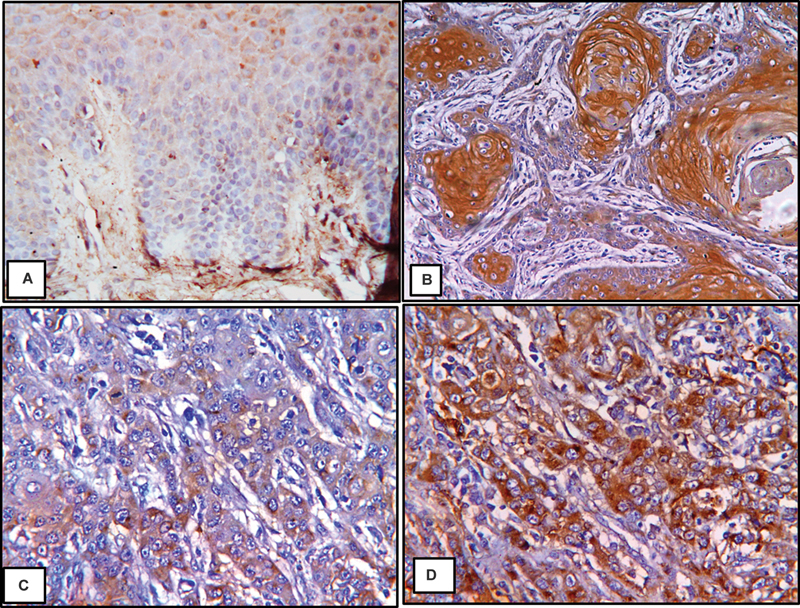
GI, normal oral epithelial tissue showing HIF-1α immunostaining was detected in cytoplasm and nucleus of all layers of the epithelium ( **A** ); GII, WDOSCC, showing HIF-1α immunostaining was seen in the cytoplasm and nucleus of the malignant cells as well as in the keratin pearls ( **B** ); GIII, MDOSCC, showing HIF-1α immunopositivity was seen in cytoplasm and nucleus of the neoplastic cells ( **C** ); and GIV, PDOSCC, showing HIF-1α immunopositivity was detected in both nucleus and cytoplasm of highly anaplastic cells ( **D** ) (HIF-1α, ×200). HIF, hypoxia-inducible factor; MDOSCC, moderately differentiated oral squamous cell carcinoma; PDOSCC, poorly differentiated oral squamous cell carcinoma; WDOSCC, well-differentiated oral squamous cell carcinoma.

Beclin-1 immunostaining was seen in the cytoplasm and nucleus of all layers of stratified squamous epithelium. Beclin-1 immunostaining was assessed in all grades of OSCC. In WDOSCC, immunopositivity was seen in both the nucleus and cytoplasm of cancer cells and the keratin of the keratin pearls. In moderately differentiated OSCC, Beclin-1 immunopositivity was seen in malignant cells' nucleus and cytoplasm. In poorly differentiated OSCC (PDOSCC), Beclin-1 immunopositivity was seen mainly in the cytoplasm of highly anaplastic cells ( Fig. 2A–D ). The normal oral epithelial tissue (GI) had the highest mean area percentage (54.66%), according to statistics. The lowest values, however, were noted in PDOSCC (GIV) (21.23%). A one-way ANOVA test revealed that *p* value was less than or equal to 0.05, so all groups showed a significant difference in between. The Tukey's post hoc test revealed a statistically significant difference between GI and G(II, III, and IV) and between GII and GIV. At the same time, there were no statistically significant differences between GII and GIII, or between GIII and GIV ( Table 1 ). As shown in Fig. 3A–D , the suprabasal cell layers' cytoplasm and nucleus of GI were found to be immunostained for HIF-1α. All OSCC grades had their HIF-1α immunostaining evaluated. In WDOSCC, the nucleus and cytoplasm of the neoplastic cells were immunopositive, as well as keratin of the epithelial and keratin pearls. HIF-1α immunopositivity was observed in the cytoplasm and nucleus of neoplastic cells in MDOSCC and PDOSCC. According to statistics, PDOSCC had the highest average area percentage at 41.48%, while normal tissue had the lowest at 14.72%. The ANOVA test has shown a statistical significant difference among all groups ( *p* < 0.000). The Tukey's post hoc test revealed a significant difference between GI and all groups of OSCC and between (GII, GIV) and (GIII, GIV). At the same time, there was no statistically significant difference between (GII, GIII) ( Table 1 ). The correlation test results showed that Beclin-1 and HIF-1α had a strong, significant negative correlation ( Table 2 ).

**Table 1:** Comparison of the mean area percentage of both Beclin-1 and HIF-1α between all groups

	Mean	Standard deviation	Standard error	95% confidence interval for mean		Minimum	Maximum
				Lower bound	Upper bound		
Beclin-1							
Group I	54.6620 ^a^	9.09017	4.06525	43.3751	65.9489	43.38	63.09
Group II	36.8040 ^b^	7.06481	3.15948	28.0319	45.5761	28.92	47.42
Group III	28.7100 ^bc^	3.74023	1.67268	24.0659	33.3541	24.98	33.59
Group IV	21.2260 ^c^	3.10286	1.38764	17.3733	25.0787	17.22	25.33
HIF-1α							
Group I	14.7200 ^a^	2.57954	1.15361	11.5171	17.9229	11.20	17.53
Group II	27.6200 ^b^	7.19829	3.21917	18.6821	36.5579	21.90	38.88
Group III	32.7020 ^b^	5.45800	2.44089	25.9250	39.4790	27.34	40.91
Group IV	41.4800 ^d^	3.92352	1.75465	36.6083	46.3517	35.62	45.99

Abbreviation: HIF, hypoxia-inducible factor.

Notes: Tukey's post hoc test means with different superscript letters are statistically significantly different.

Significance at *p* ≤ 0.05.

**Table 2:** Correlation between Beclin-1 and HIF-1α showed a strong, significant negative correlation

		Beclin-1	HIF-1α
Beclin-1	Pearson's correlation	1	−0.838 a
	Significance (two-tailed)		0.0000

Abbreviation: HIF, hypoxia-inducible factor.

a Correlation is significant at the 0.01 level (two-tailed).

## Discussion

Since OSCC is a type of solid tumor, hypoxia and starvation are common occurrences for cancer cells, leading to the potential for metabolic stress. So, it was assumed that Beclin-1 and HIF-1α had a role in the initiation and advancement of OSCC. This study aimed to estimate the prognostic significance of Beclin-1 and HIF-1α IHC expression in OSCC. Considering the Beclin-1 results for the various studied groups, Beclin-1 immunoexpression was found in normal oral epithelial tissue at the highest mean area percentage, followed by the group of WDOSCC. At the same time, PDOSCC showed the lowest mean area percentage. Autophagy, a carefully regulated process that degrades and recycles biological components, is one crucial mechanism for maintaining homeostasis. These results are in accordance with the findings published by a previous study that compared adjacent normal tissues with oral tongue squamous cell carcinoma (OTSCC) tissues. There was a reduction in Beclin-1 expression in 78.6% of cases, indicating a potential role for Beclin-1 in cancer development. Conversely, it was found that Beclin-1 expression was high in nondysplastic Barratt's esophagus cells and normal esophageal epithelial cells but low in esophageal adenocarcinomas identified by real-time polymerase chain reaction, immunoblotting, and IHC. 16 17

Based on the results of several studies, poor differentiation, lymph node metastases, and an advanced clinical TNM stage were associated with lower Beclin-1 expression by IHC, indicating a potential role for Beclin-1 in the tumor's genesis and progression. 12 16 18 On the other hand, increased Beclin-1 expression in OSCCs was associated with the development of the disease and may also stimulate tumor infiltration, 19 and depending on the cancer stage, the extent of the ischemia, and time, autophagy may have both pro- and antitumor activities. Thus, elevated Beclin-1 expression was related to the absence of lymphatic invasion, reduced cell proliferation, reduced invasiveness, and metastasis in pancreatic ductal adenocarcinoma, laryngeal carcinoma, and esophageal carcinoma. On the other hand, many studies have found that patients with reduced Beclin-1 expression show a significant overall survival benefit compared with those with higher expression as in nasopharyngeal carcinoma, ovarian carcinoma, and endometrial adenocarcinoma. These different results suggest that Beclin-1 may have distinct roles in various carcinomas; methodological variations in the assessment of IHC and the small sample size might contribute to different results. Therefore, the method of inhibiting autophagy has recently emerged to improve chemotherapy's effectiveness. This was clarified by the fact that Beclin-1-activated autophagy is essential for anticancer therapy, and the ability of cancer cells to withstand some chemotherapy drugs promotes the autophagic process, which in turn increases the ability of cancer cells to survive. 17 20

A common symptom of hypoxia is the spread of both systemic and localized cancers, as well as treatment resistance and a poor prognosis. Hypoxic tumor cells produce miR-21-rich exosomes, which are then transported to normoxic cells, where they enhance prometastatic activity. Moreover, exosomes produced by hypoxic OSCC cells promote tumor cell migration and invasion in a way dependent on HIF-1α. 21 Current research indicates that HIF-1α expression in PDOSCC had the highest mean area percentage value, while in normal oral epithelial tissues, it showed the statistically significant lowest mean area percentage. These results align with previous research that described that OSCC samples exhibit increased expression of HIF-1α protein compared with normal oral mucosa samples. 21 In our research, the mean area percentage of immunopositive carcinoma cells for HIF-1α increased from well-differentiated to poorly differentiated grade, indicating a change in HIF-1α expression corresponding to the various grades of OSCC. Our results were following another study reporting a statistically significant increase in HIF-1α expression as OSCC grade evolved from well-differentiated to poorly differentiated. 22 HIF-1α is an essential transcriptional regulator of several genes linked to adaptive cellular responses to hypoxia. Elevated HIF-1α expression has been linked to chemoradiotherapy resistance, distant metastases, and enhanced cancer cell invasion. 14 23 Sustained hypoxic conditions within tumors induce enhanced activation of the HIF-1α system, which causes specific cellular alterations that facilitate tumor growth. This leads to a clinically aggressive tumor phenotype, with a tendency toward locally invasive, distant and regional spread, and poor prognosis. 24 25

### Correlation among Beclin-1 and HIF-1α Role in OSCC

**Fig. 4:**
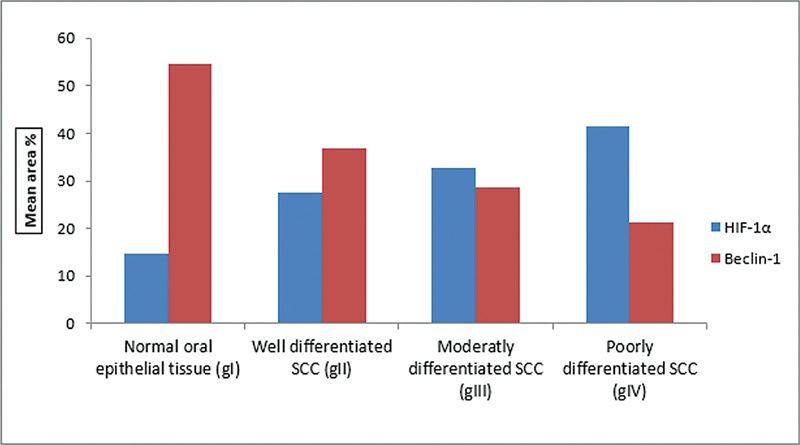
Column chart showing comparison between the mean area percentage of both Beclin-1 and HIF-1α in different groups. HIF, hypoxia-inducible factor; OSCC, squamous cell carcinoma.

**Fig. 5:**
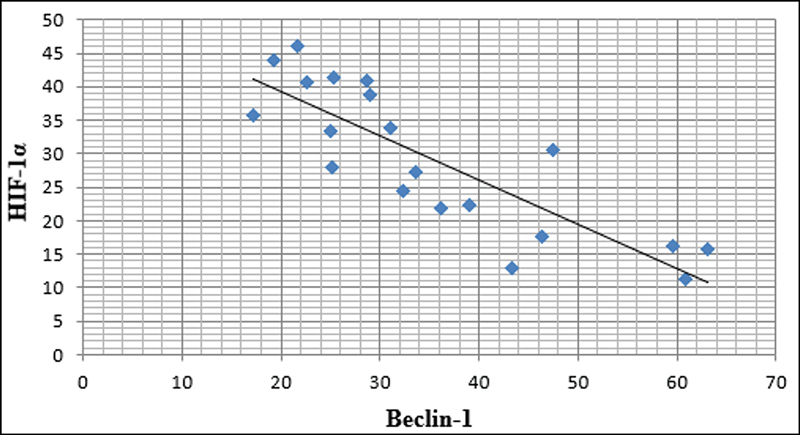
Line chart showing a strong negative correlation between Beclin-1 and HIF-1α.

It was observed that there was an inverse negative correlation between the expression of HIF-1α and Beclin-1. The correlation between reduced Beclin-1 expression and the more severe histopathological grading was more significant in the hypoxic cases of OSCC, similar to Alabiad et al 12 in serous ovarian carcinoma. Lee et al 27 discovered that the absence of Beclin-1 was associated with aggressive behavior and an increase in the formation of new blood vessels in the more oxygen-deprived areas of the tumor. This corroborates our findings about the involvement of autophagy and hypoxia in advancing OSCC ( Figs. 4 and 5 ).

Other studies have shown a positive correlation between high HIF-1α levels and Beclin-1 expression, in contrast to our results. These studies have found that cancers that overexpress HIF-1α are more likely to have elevated levels of Beclin-1 than cancers with low HIF-1α expression, as seen in nasopharyngeal and breast carcinomas. 27 28 29 One way to explain this is the variable role that hypoxia plays in the activation of autophagy in different types of cancer. Thus, our study's demonstration of the relationship between Beclin-1 and HIF-1α supports hypoxia's critical role in the induction of autophagy. Furthermore, decreased Beclin-1 and elevated levels of HIF-1α expression are associated with aggressive clinical characteristics and an unfavorable prognosis, thereby confirming their crucial involvement in the advancement, invasion, and spread of OSCC. Although the importance of integrating molecular markers into clinical practice to improve the management and prognosis of patients with OSCC, the interactions between Beclin-1 and HIF-1α and other molecular pathways are not fully understood, requiring further research to verify their prognostic role properly.

## The More Appropriate Conclusion

The strong association between Beclin-1 and HIF-1α highlights hypoxia's essential role in initiating autophagy. Furthermore, a strong significant correlation was observed between decreased Beclin-1 levels and increased HIF-1α expression, which strongly supports their crucial involvement in the initiation and progression of OSCC, as indicated by the histological grading. These findings suggest that both markers could serve as promising targets for molecular therapies in OSCC when used with existing treatment strategies.
